# Development of secretome-based strategies to improve cell culture protocols in tissue engineering

**DOI:** 10.1038/s41598-022-14115-y

**Published:** 2022-06-15

**Authors:** O. Cases-Perera, C. Blanco-Elices, J. Chato-Astrain, C. Miranda-Fernández, F. Campos, P. V. Crespo, I. Sánchez-Montesinos, M. Alaminos, M. A. Martín-Piedra, I. Garzón

**Affiliations:** 1 Department of Plastic Surgery, University Hospital Virgen de las Nieves, Granada, Spain; 2 Doctoral Program in Biomedicine, University of Granada, Granada, Spain; 3 Department of Histology (Tissue Engineering Group), Faculty of Medicine, University of Granada, Avenida de la Investigación 11, 18016 Granada, Spain; 4 Instituto de Investigación Biosanitaria Ibs.GRANADA, Granada, Spain; 5 Department of Human Anatomy and Embryology, University of Granada, Granada, Spain

**Keywords:** Medical research, Molecular medicine, Skin diseases

## Abstract

Advances in skin tissue engineering have promoted the development of artificial skin substitutes to treat large burns and other major skin loss conditions. However, one of the main drawbacks to bioengineered skin is the need to obtain a large amount of viable epithelial cells in short periods of time, making the skin biofabrication process challenging and slow. Enhancing skin epithelial cell cultures by using mesenchymal stem cells secretome can favor the scalability of manufacturing processes for bioengineered skin. The effects of three different types of secretome derived from human mesenchymal stem cells, e.g. hADSC-s (adipose cells), hDPSC-s (dental pulp) and hWJSC-s (umbilical cord), were evaluated on cultured skin epithelial cells during 24, 48, 72 and 120 h to determine the potential of this product to enhance cell proliferation and improve biofabrication strategies for tissue engineering. Then, secretomes were applied in vivo in preliminary analyses carried out on Wistar rats. Results showed that the use of secretomes derived from mesenchymal stem cells enhanced currently available cell culture protocols. Secretome was associated with increased viability, proliferation and migration of human skin epithelial cells, with hDPSC-s and hWJSC-s yielding greater inductive effects than hADSC-s. Animals treated with hWJSC-s and especially, hDPSC-s tended to show enhanced wound healing in vivo with no detectable side effects. Mesenchymal stem cells derived secretomes could be considered as a promising approach to cell-free therapy able to improve skin wound healing and regeneration.

## Introduction

Human skin is the largest organ of the body, and plays an important role in protection against external factors, also in temperature regulation, hydration, and stimulus perception^1^. Histologically, the human skin is composed of the epidermis, dermis, hypodermis, appendages, and nerve receptors^2^. Clinical disorders such as cancer, psoriasis, ichthyoses, bullous diseases, autoimmune diseases, endocrine disorders, nutritional alterations, or vascular insufficiency can disrupt the normal function and structure of the human skin^3–5^. Despite important advances in the microsurgical field in recent decades, treatment of these diseases may be challenging, and the results remain suboptimal^6,7^. In this context, tissue engineering offers a promising strategy that combines cells, extracellular matrix, and signaling molecules to improve regeneration and clinical outcomes^8,9^.

Current advances in tissue engineering are focused on the development of artificial skin substitutes based on different types of biomaterials and cell sources^10^. One of the main challenges in this field is obtaining viable epithelial cell cultures in a minimum of time. In this connection, skin keratinocytes have been reported to be relatively quiescent, and their proliferation rate is low^11^, making the biofabrication process of the skin difficult and slow^12^. For this reason, improvements are needed in epithelial cell culture methods^13^.

Several therapeutic products derived from mesenchymal stem cells (MSC) are currently being considered as enhancers of cell migration and proliferation, and it has been suggested that these products may act efficiently on slow-cycling skin epithelial cells^14^ and dermal fibroblasts. Among these products, secretomes produced by MSC contain numerous bioactive molecules that have attracted great scientific interest due to their potential to trigger phenotypic changes in cultured cells^15,16^. Secretomes are known to contain different growth factors, cytokines, proteases, transcription factors, and other molecules involved in cell adhesion, migration, proliferation, and differentiation^17^. Moreover, the bioactive molecules of secretomes are critical mediators of intercellular communication and the regulation of DNA and RNA synthesis. In addition, the nanoscale size of most secretome components can facilitate cell diffusion and permeability^18^.

However, the usefulness of different types of secretome in human skin epithelial cell cultures has not been studied to date. In addition, there is a lack of studies comparing the effects of different secretomes generated from three different types of human mesenchymal stem cells derived from the adipose tissue, dental pulp and umbilical cord on wound healing. The novelty of the present study is that we evaluated for the first time the effects of three different types of secretome derived from several types of MSC on cultured human skin epithelial cells, in order to determine the potential of these products to enhance cell proliferation and improve biofabrication strategies for tissue-engineered skin.

## Materials and methods

### Isolation and culture of human mesenchymal stem cells

To generate primary cell cultures of human MSC, we obtained samples of human adipose tissue, dental pulp, and umbilical cord according to previously described protocols^19–21^. Samples corresponding to three different donors were used (n = 3 for each tissue). Adipose tissue was used to generate human adipose-derived MSC (hADSC), whereas dental pulp MSC (hDPSC) were obtained from human teeth, and the umbilical cords were used to generate Wharton’s jelly MSC (hWJSC).

All tissue samples were digested enzymatically in a 2 mg/mL solution of *Clostridium histolyticum* type I collagenase (Gibco-Thermo Fisher Scientific, Waltham, MA, USA) for 6 h at 37 °C. Isolated cells were collected by centrifugation. hADSC and hDPSC were cultured in Dulbecco’s Modified Eagle’s Medium (DMEM) (Sigma-Aldrich/Merck, Burlington, MA, USA) supplemented with 10% fetal bovine serum (FBS) and 1% antibiotics and antimycotics (100 U/mL penicillin G, 100 mg/mL streptomycin, and 0.25 mg/mL amphotericin B) (Sigma-Aldrich/Merck). hWJSC were cultured in Amniomax-C100 culture medium (Gibco-Thermo Fisher Scientific)^21–23^. In all cases, cells were kept under standard cell culture conditions at 37 °C in a humidified incubator (Esco Lifesciences Group, Singapore, Singapore) with 5% CO_2_. Once cells reached 70% confluence, they were trypsinized with 0.25% trypsin-ethylenediaminetetraacetic acid (EDTA) (Sigma-Aldrich/Merck).

### Cell culture of human skin epithelial cells

Human skin epithelial cells (HSEC) (CRL-4048) were purchased from the American Type Culture Collection (ATCC) (Manassas, VA, USA). Cells were cultured in epithelial cell culture medium consisting of a mixture of HAM-F12 (150 mL), DMEM (300 mL) and FBS (50 mL) supplemented with penicillin/streptomycin (50 IU/mL), adenine (24 µg/mL), insulin (5 µg/mL), triiodothyronine (1.3 ng/mL), hydrocortisone (0.4 µg/mL) and epidermal growth factor (EGF) (10 mg/mL) (all from Sigma-Aldrich/Merck). The HSEC were grown at 37 °C in a humidified incubator with 5% CO_2_, and the culture medium was replaced every 2–3 days.

### Obtaining the different types of secretome, and HSEC exposure

The culture medium for each type of MSC was removed and replaced with fresh medium, and 72 h later the medium containing the products released by each type of MSC were harvested as previously reported^24,25^. Briefly, the medium was centrifuged for 10 min to eliminate all cellular debris and apoptotic bodies, followed by filtering through a sterile filter with a pore diameter of 0.22 µm (Sarstedt, Nümbrecht, Germany). Secretomes obtained from the three cultures of each type of MSC were pooled together in a single secretome suspension. Secretomes obtained from hADSC, hDPSC and hWJSC were called hADSC-s, hDPSC-s, and hWJSC-s, respectively.

To evaluate the effects of each secretome on HSEC, these cells were cultured in 96-well cell culture plates (Sarstedt) at a cell density of 1.7 × 10^4^ cells/cm^2^ with epithelial cell culture medium. 48 h later, the epithelial cell medium was removed, and cells were washed in phosphate-buffered saline (PBS) and cultured with epithelial cell medium supplemented with increasing concentrations of each type of secretome: 0%, 25% (500 µg of total protein per mL), 50% (1000 µg/mL), 75% (1500 µg/mL), 100% (2000 µg/mL). HSEC were cultured with each type and each concentration of secretome for 24, 48, 72 and 120 h.

### Protein analysis of each secretome

The presence of relevant proteins in each type of secretome (hADSC-s, hDPSC-s, hWJSC-s) and in basic nonconditioned media was determined with a human protein array kit (R&D Systems Inc., Minneapolis, MN, USA; Cat: ARY007) according to the manufacturer’s instructions. First we quantified the protein concentration in each secretome with a Coomassie brilliant blue G quantification kit (Sigma-Aldrich/Merck). Then the protein array membranes of the kit were blocked with 2.0 mL buffer, and 1.0 mL of each MSC secretome was added to a final concentration of 2000 µg total protein per mL. All samples were then treated with 15 µL reconstituted detection antibody cocktail, washed, and incubated with 2.0 mL streptavidin-HRP. Lastly, the samples were washed, and the membranes were coated with Chemi Reagent Mix and incubated for 1 min at room temperature prior to X-ray film exposure. The films were scanned, and signal intensity was quantified at each spot with Image J software (Wayne Rasband, NIH, MD, USA) in four technical replicates (n = 4). The results were normalized to total protein concentration in the controls included in the array.

### Cell viability analyses

To determine the biological effects of hADSC-s, hDPSC-s, and hWJSC-s on cell viability, a cell viability/cytotoxicity analysis kit (LIVE/DEAD Life Technologies, Carlsbad, CA, USA) was used according to a previously described method^26,27^. In brief, HSEC incubated with each type and concentration of secretome were washed in PBS, and a working solution of acetoxymethyl calcein and ethidium bromide was added for 15 min at 37 °C. The cells were then rinsed in PBS, and histological images were obtained with a Nikon Eclipse Ti-U inverted fluorescence microscope (Nikon, Tokyo, Japan). The percentage of live cells (stained green) and dead cells (stained red) was quantified in each sample. Positive controls consisted of HSEC cultured with different concentrations (25%, 50%, 75%, 100%) of basal culture medium: DMEM for hADSC and hDPSC, and Amniomax-C100 for hWJSC. In additional, negative controls consisted of HSEC treated with 2% Triton X-100 (Sigma-Aldrich/Merck). All analyses were done in triplicate (n = 3).

Cell viability was also assessed by quantifying free DNA, as previously reported^28^. Briefly, 2.0 µL was collected from cultures in each condition (i.e., HSEC cultured in the different types and concentrations of secretome at increasing follow-up times), and the amount of DNA released into the medium was quantified with UV–Vis NanoDrop 2000 equipment (Gibco-Thermo Fisher Scientific). Positive and negative controls were used as described for the LIVE/DEAD assay, and all analyses were done in triplicate (n = 3). The results were expressed as percentage cell viability after each value was normalized to the negative and positive controls.

### Cell proliferation analyses

To study the capacity of MSC secretomes to promote HSEC proliferation, each culture was followed for 24, 48, 72 and 120 h, and the number of cells was quantified at each time point with flow cytometry. Each group of cells (HSEC cultured with different types and concentrations of secretome at each time point) was rinsed twice in PBS and trypsinized with 0.25% trypsin–EDTA (Sigma/Aldrich Merck). Then the detached cells were resuspended in buffer consisting of a mixture PBS with 10% FBS and 2% EDTA (Sigma/Aldrich Merck) for flow cytometry, and the number of cells in each group was quantified with a NovoCyte Flow Cytometer (ACEA Biosciences, San Diego, CA, USA). As a positive control, HSEC cultured in epithelial cell medium were used, and the results were normalized to the control value to calculate the fold-change (FC) in the amount of cells with reference to the control group (FC = 1). All analyses were done in triplicate (n = 3).

Cell proliferation was then analyzed with the WST-1 colorimetric assay (Cell proliferation reagent WST-1, Sigma/Aldrich Merck) as previously reported^28,29^. Briefly, water-soluble tetrazolium salt was added to the culture medium of each study group (HSEC cultured with the different types and concentrations of secretome at different time points), and cells were incubated in this mixture for 4 h at 37 °C. Then colorimetric analysis was done by measuring absorbance at 450 nm with an Asys UVM-340 microplate spectrophotometer reader (Biochrom, Cambridge, UK). Positive and negative controls were used as described for cell viability analyses, and the results were normalized to positive controls (considered 100%) and negative controls (considered 0%). All analyses were done in triplicate (n = 3).

### In vitro wound healing test

To determine the ability of HSEC in each study group to repair a tissue defect, ex vivo wound healing analysis was carried out with the Oris Universal Cell Migration Assembly Kit (Platypus Technologies, Fitchburg, WI, USA). HSEC cells were cultured at a cell density of 17,000 cells/cm^2^ in 96-well plates containing the stoppers provided by the manufacturer. Epithelial cell medium was used for 48 h to favor cell confluence. Then the stoppers were physically removed from the plates containing confluent cells to allow the cells to migrate to areas previously occupied by the stoppers, and the epithelial culture medium was replaced by each type and concentration of secretome (25%, 50%, 75%, 100%). In order to perform a fluorescent analysis to evaluate cell migration, 1 μL of a 200 mM calcein solution was added to each well and images were obtained after 12, 24 and 36 h of follow-up with a Nikon Eclipse Ti-U inverted microscope. These analyses were performed in triplicate (n = 3). In each sample, the area occupied by HSEC and the empty surface area devoid of cells were quantified, and the results were normalized to the control at time 0 (considered 100% empty space).

### Preliminary in vivo wound healing analysis

To evaluate the in vivo effects of the different secretomes, each type of secretome was applied on full-thickness skin injuries inflicted in Wistar laboratory rats. All animals used in the present work were adult 12-week-old male rats, and experiments were carried out using sextuplicates (n = 6). Each animal was deeply anesthetized with ketamine and acepromazine. Then, an 8 mm dermatological punch was used to generate 4 identical circular injuries at the back of each animal, by removing the skin excised by the dermatological punch. If a small hemorrhage was generated by the procedure, hemostasis was achieved by mechanically pressuring the injury for 2–3 min with a sterile gauze. Then, 50 µL of each type of secretome were applied to each skin defect (control medium, hADSC-s, hDPSC-s or hWJSC-s, respectively) using a micropipette. Control medium consisted of DMEM with 10% FBS and 1% antibiotics and antimycotics, since secretomes were generated and diluted in the same medium. The skin defects were then protected with a sterile gauze and animals were followed-up for 4 weeks, with application of each type of secretome every 24 h. Images of each defect were taken after 4 weeks, and the area occupied by each defect was automatically quantified using the area fraction option of the ImageJ software (National Institutes of Health, Bethesda, MD, USA) after converting the image into binary (black and white).

Animals were euthanatized after the follow-up period of 4 weeks, and the regeneration areas were surgically extracted and fixed in formalin for histological analysis. In brief, fixed tissues were embedded in paraffin and histological sections were obtained. Sections were deparaffinized and stained with hematoxylin–eosin and Masson trichrome staining (for global morphology), picrosirius red (for collagen fibers) and alcian blue (for proteoglycans) using routine histological and histochemical methods previously described^8,9,12,20^.

### Statistical analysis

The Kruskal–Wallis test was used to detect overall differences among all groups used in this study. Thereafter, specific pairwise comparisons were done with the Mann–Whitney post-hoc test. This statistical analysis was used to compare each study group (each type of secretome, each concentration, and each time point) with the control group for each study group (hADSC-s and hDPSC-s vs. DMEM, and hWJSC vs. Amniomax C-100). The same test was used to compare the results obtained for two different types of secretome in the protein analysis tests, cell viability, and cell proliferation analyses, as well as to compare the size of the defects generated in laboratory animals. Correlations between two test results were checked with Spearman’s rho test. All statistical tests were done with SPSS v. 25 software (SPSS, Inc., Chicago, IL, USA) and with the Excel Real Statistics add-in, available at http://www.real-statistics.com (Dr. Charles Zaiontz, Purdue University, West Lafayette, IN, USA). The significance level was set at 5% for all tests.

### Ethics declarations

The study was conducted according to the guidelines of the Declaration of Helsinki, and all methods were carried out in accordance with relevant guidelines and regulations. The study was approved by the Institutional Research and Ethics Committee in Biomedical Research of Andalusia (Comité Coordinador de Ética de Investigación Biomédica) with protocol code S1900527, date of approval December, 27th, 2019. Informed consent was obtained from all subjects involved in the study or their legal guardians.

Animal experimentation was approved by the Animal Experimentation Ethics Committee of Granada (Comité de Ética y Experimentación Animal, CEEA) and Consejería de Agricultura, Ganadería, Pesca y Desarrollo Sostenible, Junta de Andalucía, Spain, protocol code 19/04/2021/053, date of approval April, 21st, 2021. All animal experiments were performed in accordance with relevant named guidelines and regulations and authors complied with the ARRIVE guidelines.

## Results

### Protein analysis of each type of secretome

**Figure 1:**
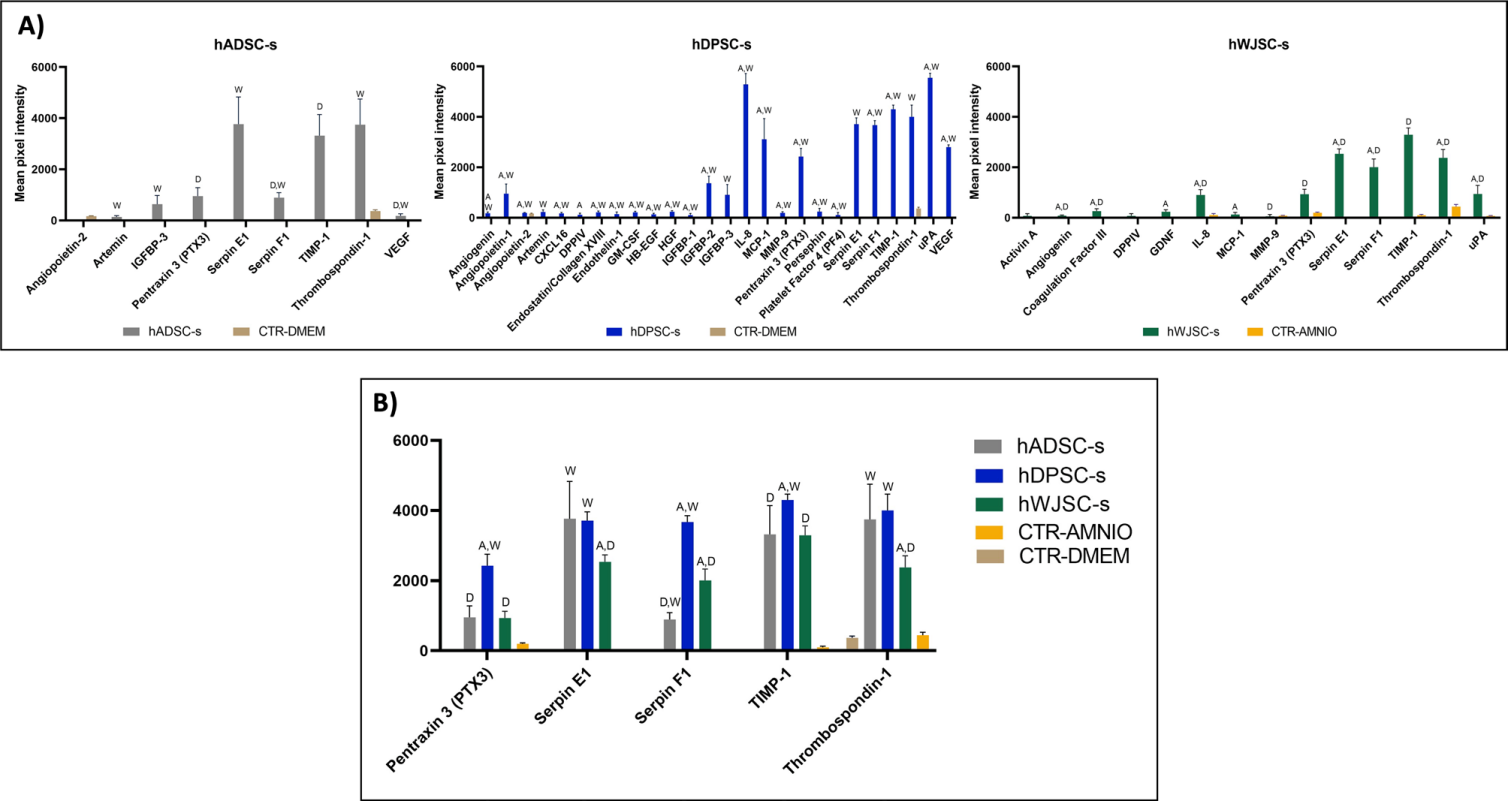
Protein analysis of each type of secretome. Values correspond to mean pixel intensity ± standard deviation for each type of secretome. *hADSC-s* human adipose-derived MSC secretome, *hDPSC-s* human dental pulp MSC secretome, *hWJSC-s* Wharton’s jelly MSC secretome. Statistically significant differences compared to hADSC-s are labeled A, differences compared to hDPSC-s are labeled D, and differences compared to hWJSC-s are labeled W.

The protein analysis of the different secretomes studied here revealed the expression of numerous relevant proteins in a secretome-dependent manner, with control media (devoid of secretomes) showing very low or no expression of the proteins found in conditioned media (Fig. 1 and Table 1). In hADSC-s, positive expression was seen for 8 out of the 55 proteins analyzed (14.54%), including VEGF, TIMP-1, thrombospondin-1, serpin E1, IGFBP-3, serpin F1, and pentraxin 3 (PTX3). Statistically significant differences were found when the expression level of all proteins was compared with hWJSC-s, except for PTX3 and TIMP-1, whereas comparison with hDPSC-s showed significant differences for VEGF, TIMP-1, serpin F1 and PTX3. In hDPSC-s, detectable levels were found for 27 of the 55 proteins included in the array (49.09%), including angiogenin, angiopoietin-1, angiopoietin-2, artemin, CXCL16, DPPIV, endostatin/collagen XVIII, endothelin-1, GM-CSF, HB-EGF, HGF, IGFBP-1, IGFBP-2, IGFBP-3, IL-8, MCP-1, MMP-9, PTX3, persephin, platelet factor 4 (PF4), serpin E1, serpin F1, thrombospondin-1, TIMP-1, uPA and VEGF, with 25 of these proteins showing statistically significant differences compared to hWJSC-s and 22 proteins significantly different to hADSC-s. When hWJSC-s was analyzed, we found positive signals for 14 proteins (25.45%), including activin A, angiogenin, coagulation factor III, DPPIV, GDNF, IL-8, MCP-1, MMP-9, PTX3, serpin E1, serpin F1, thrombospondin-1, TIMP-1 and uPA, with significant differences compared to hADSC-s for 9 of these proteins, and significant differences compared to hDPSC-s for 12 of these proteins. Interestingly, all three types of secretome showed positive expression of pentraxin-3, serpin-E1, serpin-F1, TIMP-1, and thrombospondin-1, which are proteins that play a role in cell proliferation, with hDPSC-s tending to show the highest expression of these five proteins. As expected, control nonconditioned media contained very low amounts of these five proteins.

**Table 1:** Quantification of proteins found in each type of secretome. Values correspond to mean pixel intensity ± standard deviation of each target protein. *hADSC-s* human adipose-derived MSC secretome. *hDPSC-s* human dental pulp MSC secretome, *hWJSC-s* Wharton’s jelly cell secretome. A: Differences compared to hADSC-s are statistically significant; D: Differences compared to hDPSC-s are statistically significant; W: Differences compared to hWJSC-s are statistically significant.

Target	hADSC-s	hDPSC-s	hWJSC-s
Activin A	0.00 ± 0.00	0.00 ± 0.00	74.28 ± 85.89
Angiogenin	0.00 ± 0.00^D,W^	175.98 ± 50.25^A,W^	80.53 ± 25.70^A,D^
Angiopoietin-1	0.00 ± 0.00^D^	950.81 ± 388.12^A,W^	0.00 ± 0.00^D^
Angiopoietin-2	0.00 ± 0.00^D^	193.18 ± 14.76^A,W^	0.00 ± 0.00^D^
Artemin	137.43 ± 52.20^W^	227.09 ± 86.56^W^	0.00 ± 0.00^A,D^
Coagulation factor III	0.00 ± 0.00^W^	0.00 ± 0.00^W^	262.26 ± 94.45^A,D^
CXCL16	0.00 ± 0.00^D^	172.64 ± 38.90^A,W^	0.00 ± 0.00^D^
DPPIV	0.00 ± 0.00^D^	115.05 ± 67.85^A^	73.85 ± 90.55
Endostatin/Coll XVIII	0.00 ± 0.00^D^	211.40 ± 56.44^A,W^	0.00 ± 0.00^D^
Endothelin-1	0.00 ± 0.00^D^	143.96 ± 81.59^A,W^	0.00 ± 0.00^D^
GDNF	0.00 ± 0.00^W^	0.00 ± 0.00^W^	237.52 ± 82.02^A,D^
GM-CSF	0.00 ± 0.00^D^	211.58 ± 44.20^A,W^	0.00 ± 0.00^D^
HB-EGF	0.00 ± 0.00^D^	131.00 ± 45.69^A,W^	0.00 ± 0.00^D^
HGF	0.00 ± 0.00^D^	235.84 ± 46.21^A,W^	0.00 ± 0.00^D^
IGFBP-1	0.00 ± 0.00^D^	103.67 ± 70.65^A,W^	0.00 ± 0.00^D^
IGFBP-2	0.00 ± 0.00^D^	1.367.16 ± 281.70^A,W^	0.00 ± 0.00^D^
IGFBP-3	635.52 ± 341.84^W^	908.30 ± 407.36^W^	0.00 ± 0.00^A,D^
IL-8	0.00 ± 0.00^D,W^	5.292.86 ± 431.42^A,W^	902.98 ± 209.65^A,D^
MCP-1	0.00 ± 0.00^D,W^	3.114.89 ± 816.07^A,W^	128.31 ± 82.31^A,D^
MMP-9	0.00 ± 0.00^D^	194.32 ± 44.77^A,W^	58.67 ± 67.94^D^
Pentraxin 3 (PTX3)	950.49 ± 329.62^D^	2.428.13 ± 324.78^A,W^	932.59 ± 195.52^D^
Persephin	0.00 ± 0.00^D^	242.11 ± 130.05^A,W^	0.00 ± 0.00^D^
Platelet factor 4	0.00 ± 0.00^D^	106.33 ± 101.32^A,W^	0.00 ± 0.00^D^
Serpin E1	3.766.56 ± 1.064.20^W^	3.712.11 ± 251.93^W^	2.536.60 ± 197.68^A,D^
Serpin F1	890.28 ± 195.87^D,W^	3.670.35 ± 181.96^A,W^	2.007.66 ± 327.05^A, D^
TIMP-1	3.319.92 ± 823.43^D^	4.304.53 ± 165.44^A,W^	3.291.71 ± 268.86^D^
Thrombospondin-1	3.749.86 ± 1.003.73^W^	4.002.02 ± 463.98^W^	2.376.19 ± 329.89^A,D^
uPA	0.00 ± 0.00^D,W^	5,551.01 ± 180.03^A,W^	944.53 ± 335.50^A,D^
VEGF	175.00 ± 86.53^D,W^	2,800.51 ± 92.91^A,W^	0.00 ± 0.00^A,D^

### Cell viability analysis

**Figure 2:**
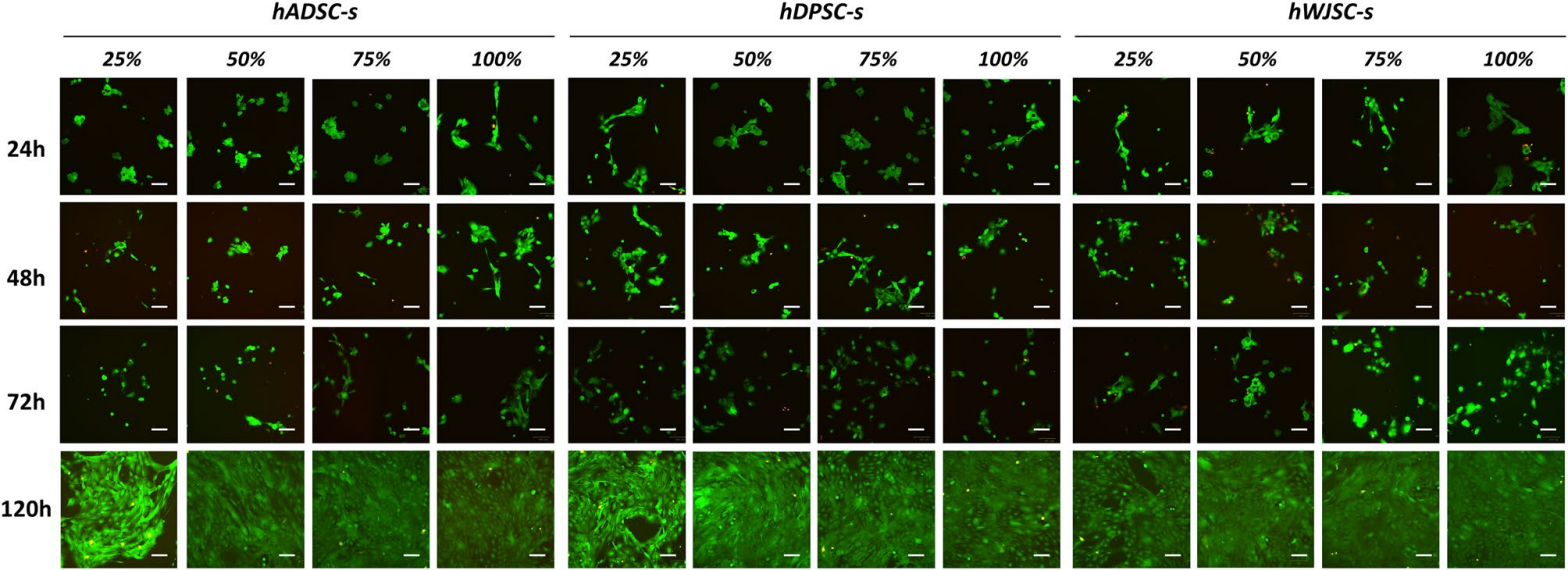
Analysis of cell viability as determined by LIVE/DEAD assay in HSEC exposed to different types (hADSC-s, hDPSC-s and hWJSC-s) and concentrations (25%, 50%, 75%, 100%) of secretome after 24, 48, 72 and 120 h of follow-up. Live cells are stained green whereas dead cells are stained red. Scale bars: 100 µm. *hADSC-s* human adipose-derived MSC secretome, *hDPSC-s* human dental pulp MSC secretome, *hWJSC-s* Wharton’s jelly MSC secretome.

We first analyzed the biosafety of secretome treatment by determining cell viability with the LIVE/DEAD assay. The results showed that the use of secretomes was safe for HSEC, and viability was high in all study groups (Fig. 2, Table 2, and Supplementary Table 1).

**Table 2:** Quantitative analysis of cell viability as determined by LIVE/DEAD assay and DNA quantification in HSEC exposed to different types (hADSC-s, hDPSC-s and hWJSC-s) and concentrations (25%, 50%, 75%, 100%) of secretome, or to control (CTR) nonconditioned medium (AMNIOMAX C100 or DMEM) after 24, 48, 72 and 120 h of follow-up. Values are shown as averages ± standard deviations. For the LIVE/DEAD assay, values correspond to percentages of live cells in each group. For DNA quantification, values correspond to the amount of DNA in ng/µL. *hADSC-s* human adipose-derived MSC secretome, *hDPSC-s* human dental pulp MSC secretome, *hWJSC-s* Wharton’s jelly MSC secretome.

Viability	LIVE/DEAD assay				DNA quantification			
	24 h	48 h	72 h	120 h	24 h	48 h	72 h	120 h
**hADSC-s**								
25%	92.54 ± 0.94	92.7 ± 0.9	91.35 ± 2.32	97.11 ± 0.49	260.94 ± 3.51	275.6 ± 5.23	277.67 ± 3.73	245.12 ± 1.87
50%	87.87 ± 3.51	90.6 ± 2.96	86.45 ± 2.79	99.11 ± 0.21	264.69 ± 11.82	296.89 ± 30.47	282.75 ± 5.32	234.42 ± 4.97
75%	85.82 ± 4.45	94.35 ± 1.8	87.2 ± 5.22	99.26 ± 0.16	251.33 ± 11.22	259.59 ± 15.39	255.76 ± 17.66	226.72 ± 5.73
100%	87.34 ± 4.31	93.5 ± 0.61	92.17 ± 1.73	99.21 ± 0.16	236.19 ± 1.96	237.19 ± 4.7	232.03 ± 0.8	228.19 ± 11.1
**hDPSC-s**								
25%	90.55 ± 1.9	95.17 ± 0.6	93.53 ± 0.86	97.11 ± 1.73	226.97 ± 1.06	226.97 ± 1.06	226.6 ± 2.14	208.69 ± 24.88
50%	91.07 ± 0.98	95.33 ± 1.9	90.15 ± 6.21	99.09 ± 0.24	199.29 ± 19.77	199.29 ± 19.77	212.42 ± 32.26	168.71 ± 7.45
75%	94.73 ± 0.5	93.96 ± 1.81	93.09 ± 3.21	98.59 ± 0.27	184.45 ± 6.22	184.45 ± 6.22	185.66 ± 15.36	167.12 ± 2.64
100%	91.85 ± 2.19	96.21 ± 0.96	93.87 ± 0.55	97.99 ± 0.33	173.34 ± 16.44	193.19 ± 34.08	152.03 ± 1.6	182.04 ± 7.78
**hWJSC-s**								
25%	86.58 ± 1.89	95.14 ± 1.75	94.51 ± 2.54	99.06 ± 0.39	241.64 ± 3.92	241.2 ± 2.91	242.24 ± 7.05	228.12 ± 8.56
50%	89.28 ± 0.99	93.1 ± 0.89	89.29 ± 7.61	98.84 ± 0.28	251.72 ± 14.57	268.57 ± 25.8	246.4 ± 0.57	227.73 ± 20.54
75%	90.58 ± 3.76	90.54 ± 3.74	92.94 ± 3.12	99 ± 0.22	255.85 ± 9.7	258.3 ± 13.58	240.46 ± 4.32	223.91 ± 7.29
100%	89.1 ± 1.19	94.45 ± 1.64	92.23 ± 2.06	98.6 ± 0.48	238.96 ± 3.79	246.79 ± 14.59	232.38 ± 0.53	235.11 ± 5.21
**CTR-DMEM**								
25%	99.41 ± 0.42	97.89 ± 0.74	99.33 ± 0.06	99.78 ± 0.08	242.23 ± 8.65	199.7 ± 3.94	248.26 ± 31.42	235.53 ± 16.91
50%	99.45 ± 0.07	98.2 ± 0.38	99.33 ± 0.06	99.92 ± 0.02	213.58 ± 1.19	175.16 ± 6.8	213.59 ± 5.35	207.75 ± 9.13
75%	99.78 ± 0.1	98.07 ± 0.38	99.33 ± 0.06	99.91 ± 0.01	183.73 ± 14.7	174.8 ± 14.24	207.76 ± 3.42	201.19 ± 7.63
100%	99.68 ± 0.21	98.28 ± 0.47	99.33 ± 0.06	99.95 ± 0.01	188.37 ± 3.27	169.86 ± 3.18	213.7 ± 2.01	222.5 ± 5.94
**CTR-AMNIOMAX**								
25%	99.38 ± 0.13	98.6 ± 0.07	99.65 ± 0.12	99.83 ± 0.04	283.23 ± 5.21	236.27 ± 9.25	256.24 ± 15.64	242.25 ± 11.49
50%	99.69 ± 0.2	99.07 ± 0.19	99.8 ± 0.05	99.85 ± 0.07	348.81 ± 53.6	245.98 ± 4.61	282.11 ± 11.36	244.24 ± 18.96
75%	99.71 ± 0.12	99.62 ± 0.31	99.67 ± 0.16	99.66 ± 0.08	340.24 ± 2.29	261.51 ± 1.52	297.93 ± 15.23	278.65 ± 23.71
100%	99.65 ± 0.32	99.15 ± 0.08	99.76 ± 0.08	99.5 ± 0.49	398.69 ± 35.94	309.25 ± 11.14	375.29 ± 16.1	408.2 ± 68.83
**CTR-NEG**	–				468.8 ± 2.36	506.03 ± 6.55	596.63 ± 10.04	608.7 ± 3.29

**Figure 3:**
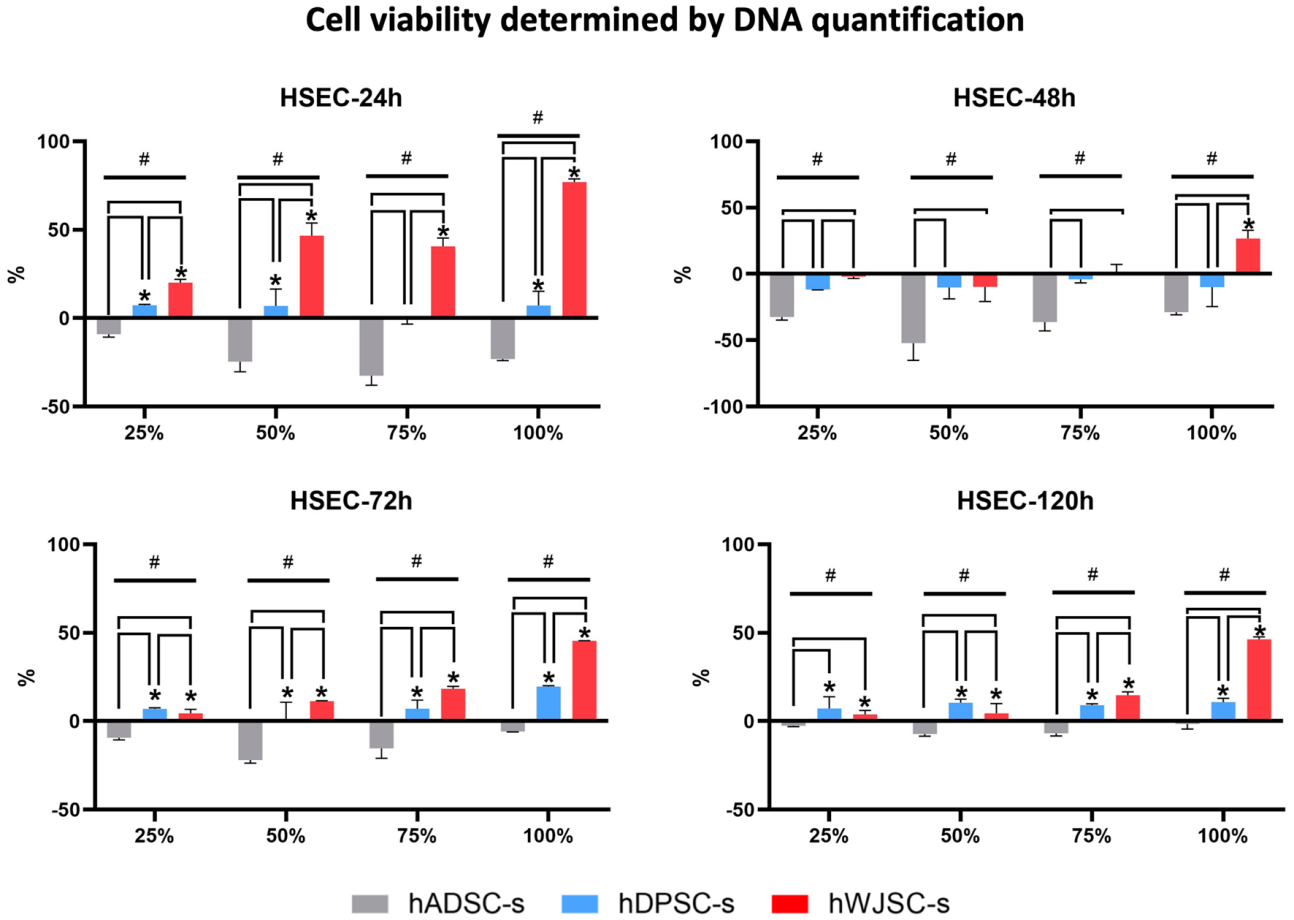
Analysis of cell viability as determined by DNA quantification in HSEC exposed to different types (hADSC-s, hDPSC-s and hWJSC-s) and concentrations (25%, 50%, 75%, 100%) of secretome after 24, 48, 72 and 120 h of follow-up. Results were normalized with respect to positive controls (in the 0 line/row) and are expressed as the percentage increase in cell viability compared to positive controls. Standard deviations are shown as error bars. *hADSC-s* human adipose-derived MSC secretome, *hDPSC-s* human dental pulp MSC secretome, *hWJSC-s* Wharton’s jelly MSC secretome. Statistically significant increases compared to controls are labeled with an asterisk (*), and comparisons between types of secretome are grouped with a horizontal square bracket. Statistically significant differences detected with the Kruskal–Wallis test are labeled with #.

Next, cell viability in HSEC cultured with the different types of secretome (hADSC-s, hDPSC-s and hWJSC-s) was evaluated by quantifying DNA released into the culture medium. The results showed that the use of specific types and concentrations of secretome were associated with improved cell viability. Some study groups showed significantly lower DNA release, and therefore higher cell viability, than control HSEC cultured in basal media. As shown in Fig. 3 and Table 2, the highest levels of cell viability were found in cells cultured with WJSC-s, followed by hDPSC-s, especially at the highest concentrations of secretome. Statistically significant differences were found between hWJSC-s and controls at all concentrations at 24, 72 and 120 h, and at a concentration of 100% at 48 h. In addition, statistically significant differences were found between hDPSC-s and controls at the same times and concentrations, except for 75% at 24 h and 100% at 48 h. Kruskal–Wallis tests disclosed overall differences among all groups at all three follow-up times analyzed for cell viability (Supplementary Table 1).

### Cell proliferation analysis

**Figure 4:**
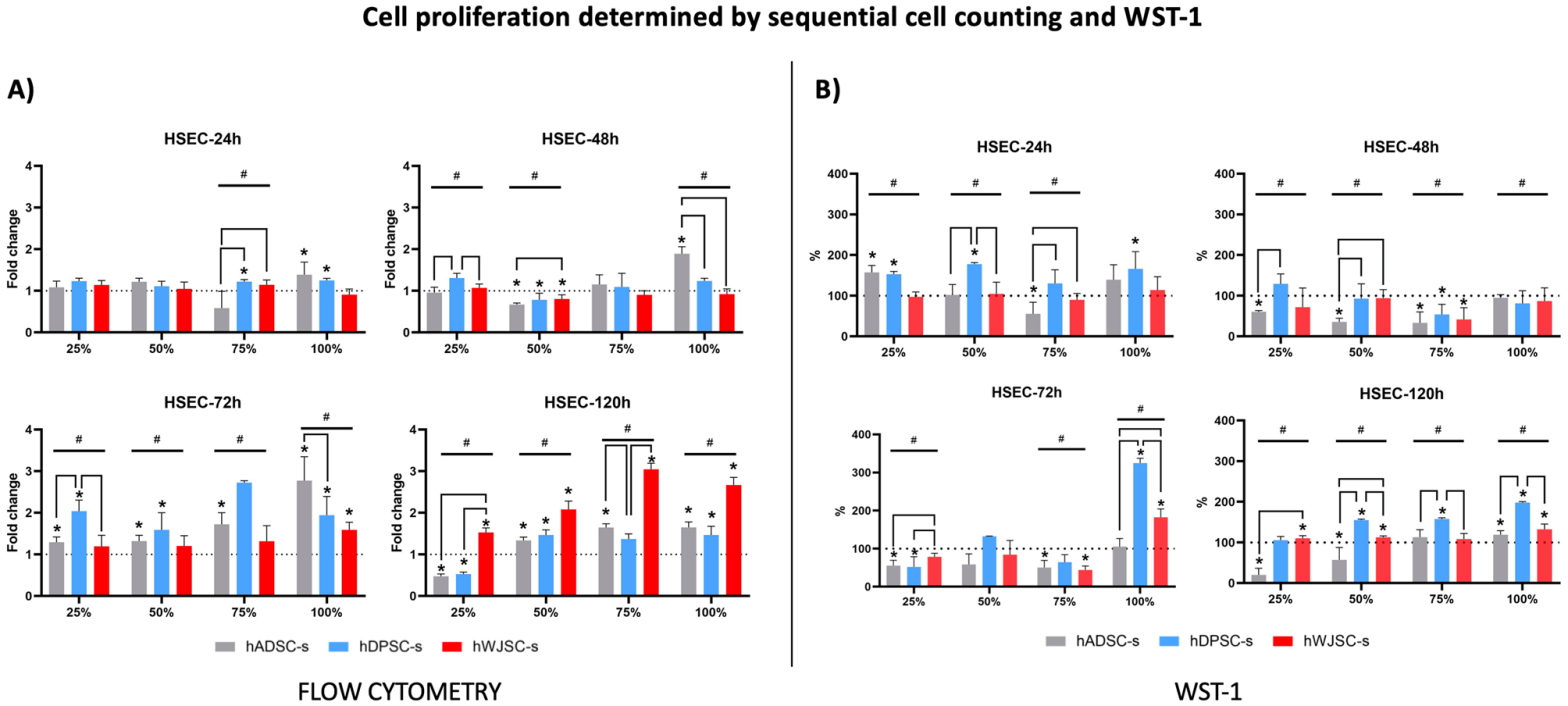
Analysis of cell proliferation determined by sequential cell counting and WST-1 analysis in HSEC exposed to different types (hADSC-s, hDPSC-s and hWJSC-s) and concentrations (25%, 50%, 75%, 100%) of secretome after 24, 48, 72 and 120 h of follow-up. (**A**) Cell counts were determined by flow cytometry. Results are shown as fold-change (FC) number relative to controls (considered FC = 1). (**B**) WST-1 activity. Results are shown as the percentage of metabolic activity after normalization with respect to controls (considered 100%). In both cases, error bars correspond to standard deviations. Statistically significant differences with controls are labeled with asterisks (*), whereas comparisons between types of secretome are grouped with a horizontal square bracket. Statistically significant differences detected with the Kruskal–Wallis test are labeled with #.

To determine the effect of the different types of secretome (hADSC-s, hDPSC-s and hWJSC-s) on cell proliferation, we first quantified the cells found at each time point with flow cytometry. Our results showed that in HSEC cultured under specific conditions, cell numbers increased significantly after 24, 48, 72 and 120 h of follow-up. As shown in Fig. 4A and Table 3, we found that the highest values were normally found at 120 h, with a significant increase compared to controls for most groups. Kruskal–Wallis tests revealed overall differences among groups and for most of the conditions tested here, except for 25%, 50% and 100% concentrations after 24 h and the 75% concentration after 48 h (Supplementary Table 1). For cells cultured with hADSC-s, we found a significant increase in cell numbers at all time points when the 100% concentration was used, with an increase of up to threefold at 72 h. When the concentration was 75%, we found significant increases only at 72 and 120 h. Similarly, a 50% concentration of hADSC-s significantly increased cell numbers at 72 and 120 h, and a 25% concentration led to a significant increase at 72 h. Interestingly, we also found a decrease in cell numbers at 48 h of follow-up with the 50% concentration, and a decrease at 120 h when the 25% concentration was used. When hDPSC-s was evaluated, we found a significantly positive effect after 24, 72 and 120 h of culture with the 100% concentration of this secretome, and after 24 h of culture with the 75% concentration. In addition, the 50% concentration was significantly associated with increases in cell numbers at 72 and 120 h, and the 25% concentration was associated with an increase at 72 h. For hWJSC-s, our results showed a significantly positive effect on cell numbers with the 100% concentration at 72 h of follow-up, and with all concentrations at 120 h.

**Table 3:** Analysis of cell proliferation as determined by sequential cell counting with flow cytometry and WST-1 activity in HSEC exposed to different types (hADSC-s, hDPSC-s and hWJSC-s) and concentrations (25%, 50%, 75%, 100%) of secretome, or to control (CTR) nonconditioned medium (AMNIOMAX-C100 or DMEM) after 24, 48, 72 and 120 h of follow-up. Results are shown as averages ± standard deviations.

Proliferation	Cell count (flow cytometry)				WST-1			
	24 h	48 h	72 h	120 h	24 h	48 h	72 h	120 h
**hADSC-s**								
25%	1010 ± 138.59	829.33 ± 109.77	744 ± 71.02	6507.33 ± 789.08	0.46 ± 0.05	0.26 ± 0.01	0.33 ± 0.08	0.28 ± 0.22
50%	1037.33 ± 73.33	682.67 ± 37.22	820.67 ± 85.66	18,955.33 ± 1135.92	0.34 ± 0.09	0.18 ± 0.05	0.37 ± 0.17	0.83 ± 0.44
75%	556 ± 385	1120 ± 225.95	980 ± 159.85	22,412 ± 1197.08	0.16 ± 0.08	0.14 ± 0.12	0.26 ± 0.1	1.6 ± 0.26
100%	1310 ± 284.02	1402.67 ± 127.9	1231.33 ± 253.51	20,658.67 ± 1655.21	0.41 ± 0.11	0.45 ± 0.04	0.69 ± 0.14	1.32 ± 0.11
**hDPSC-s**								
25%	1151.33 ± 62.68	1130 ± 98	1172 ± 152.83	7250 ± 644.88	0.45 ± 0.02	0.54 ± 0.1	0.31 ± 0.16	1.45 ± 0.12
50%	946 ± 101.82	800.67 ± 163.64	988 ± 256.49	20,780 ± 1743.05	0.6 ± 0.01	0.46 ± 0.18	0.83 ± 0	2.24 ± 0.03
75%	1162.67 ± 47.26	1064 ± 315.26	1551 ± 26.87	18,636 ± 1685.74	0.39 ± 0.1	0.23 ± 0.11	0.34 ± 0.1	2.23 ± 0.04
100%	1178.67 ± 49.17	918.67 ± 44.96	862 ± 199.19	18,373.33 ± 2650.75	0.49 ± 0.12	0.38 ± 0.15	2.13 ± 0.08	2.2 ± 0.02
**hWJSC-s**								
25%	1139.33 ± 107	1087.33 ± 97.35	842.67 ± 186.36	21,865.33 ± 1578.82	0.47 ± 0.06	0.36 ± 0.23	0.55 ± 0.07	1.53 ± 0.09
50%	1023.33 ± 157.5	853.33 ± 108.49	760.67 ± 155.35	22,395.33 ± 2135.12	0.44 ± 0.12	0.54 ± 0.12	0.54 ± 0.24	1.63 ± 0.05
75%	1194 ± 121.7	864 ± 94.32	838.67 ± 236.92	22,972.67 ± 1094.74	0.4 ± 0.07	0.23 ± 0.16	0.37 ± 0.09	1.51 ± 0.19
100%	1166.67 ± 174.82	898.67 ± 120.69	982 ± 112.8	21,572.67 ± 1472.08	0.47 ± 0.13	0.57 ± 0.21	1.1 ± 0.13	1.45 ± 0.15
**CTR-DMEM**								
25%	932.53 ± 369.51	865.38 ± 378.67	574.92 ± 93.38	13,745.59 ± 1122.3	0.35 ± 0.04	0.57 ± 0.04	0.83 ± 0.08	2.06 ± 0.03
50%	852.64 ± 219.44	1020.28 ± 30.77	621.24 ± 29.39	14,176.62 ± 1028.57	0.4 ± 0.05	0.67 ± 0.15	0.89 ± 0.13	2.16 ± 0.03
75%	952.97 ± 24.86	972.44 ± 31.32	569.16 ± 26.91	13,608.81 ± 904.44	0.35 ± 0.05	0.58 ± 0.04	0.74 ± 0.27	2.12 ± 0.02
100%	944.45 ± 42.25	742.39 ± 126.52	443.97 ± 43.9	12,541.01 ± 1053.99	0.35 ± 0	0.63 ± 0.03	0.93 ± 0.09	1.66 ± 0.05
**CTR-AMNIOMAX**								
25%	999.14 ± 47.55	1017.47 ± 48.97	705.54 ± 29.58	14,336.87 ± 1304.03	0.57 ± 0.07	0.67 ± 0.09	0.99 ± 0.09	2.07 ± 0.04
50%	976.89 ± 101.47	1063.13 ± 15.64	631.59 ± 148.71	10,756.24 ± 483.42	0.49 ± 0.02	0.78 ± 0.06	0.91 ± 0.09	2.16 ± 0.07
75%	1045.61 ± 59.26	957.74 ± 65.6	637.27 ± 92.56	7548.44 ± 1389.87	0.53 ± 0.03	0.74 ± 0.06	1.18 ± 0.19	2.08 ± 0.07
100%	1286.42 ± 508.72	975.11 ± 175.27	617.49 ± 63.47	8084.3 ± 1250.03	0.49 ± 0.01	0.88 ± 0.14	0.85 ± 0.15	1.64 ± 0.25

We then analyzed cell proliferation with WST-1 assays (Fig. 4B and Table 3). These results confirmed that certain conditions were able to induce cell proliferation in culture. When HSEC were exposed to hADSC-s, we found a significant increase in WST-1 activity with the 25% concentration at 24 h, and with the 100% concentration at 120 h, although a significant decrease was also seen under certain conditions. Culture in the presence of hDPSC-s showed a significantly positive effect on proliferation with 25%, 50% and 100% concentrations at 24 h, 100% at 72 h, and with 50%, 75% and 100% concentrations at 120 h. Cells cultured with hWJSC-s showed a significant increase in cell proliferation with the 100% concentration at 72 h, and with the 25%, 50% and 100% concentrations at 120 h. Kruskal–Wallis tests disclosed overall differences between most experimental conditions (Supplementary Table 1).

Analysis of the correlation between the flow cytometry findings and WST-1 activity showed that these two factors were positively correlated (r = 0.432; *p* < 0.001).

### In vitro wound healing analysis

**Figure 5:**
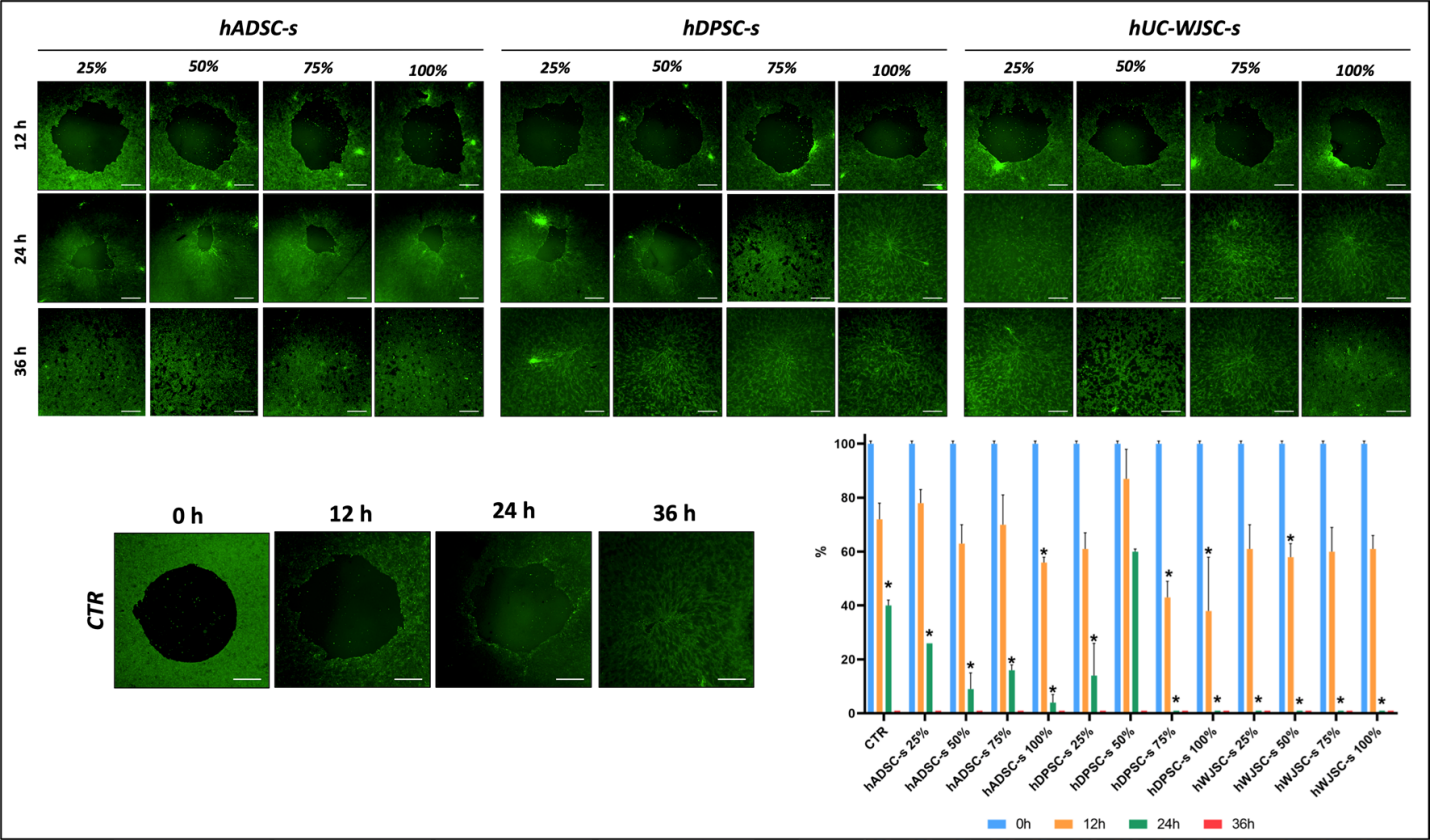
Results of wound healing fluorescent analysis of HSEC exposed to different types (hADSC-s, hDPSC-s and hWJSC-s) and concentrations (25%, 50%, 75%, 100%) of secretome after 12, 24 and 36 h of follow-up. *CTR* control group, *hADSC-s* human adipose-derived MSC secretome, *hDPSC-s* human dental pulp MSC secretome, *hWJSC-s* Wharton’s jelly MSC secretome. Scale bar: 100 μm. The histogram illustrates the results of quantification of the empty areas on each culture surface, normalized to the area at time zero (t0). Asterisks indicate statistically significant differences compared to controls analyzed at the same follow-up time.

To determine the ability of HSEC to repair a tissue defect under different secretome conditions, wound healing analysis was done at 0, 12, 24 and 36 h, until the culture surface was completely covered by confluent cells (Fig. 5). The results showed that control cells cultured in basal medium tended to proliferate and covered the entire culture surface after 36 h. For cells cultured in hADSC-s, surface coverage with the 25%, 50% and 75% concentrations of hADSC-s was similar to that seen in control cultures after 12 h. However, 100% concentrations of this secretome were associated with a significant decrease in empty surface area (*p* = 0.0286). After 24 h, a considerable percentage of the culture surface was covered by cells at all concentrations of hADSC-s, especially the highest concentrations of secretome, and statistically significant differences compared to control cells were found for all concentrations (*p* = 0.0286). At 36 h, the entire surface was covered by cells, as seen in control cultures (*p* > 0.05).

In cells cultured for 12 h in hDPSC-s, we found that the 75% and 100% secretome concentrations were able to induce cells to cover larger surface areas than control cultures, with statistically significant differences (*p* = 0.0286). At 24 h, hDPSC-s concentrations of 25%, 75% and 100% resulted in a significant surface area decrease compared to controls, and the entire surface was covered by cells after 36 h, as in control cultures (*p* > 0.05). Lastly, cells cultured with hWJSC-s for 12 h showed statistically significant differences compared to controls only with the 50% concentration of secretome. However, cells covered the entire culture surface at all concentrations of hWJSC-s after 24 h and 36 h, with statistically significant differences compared to controls (*p* = 0.0286) after 24 h of follow-up (Fig. 5).

### Preliminary in vivo analysis

**Figure 6:**
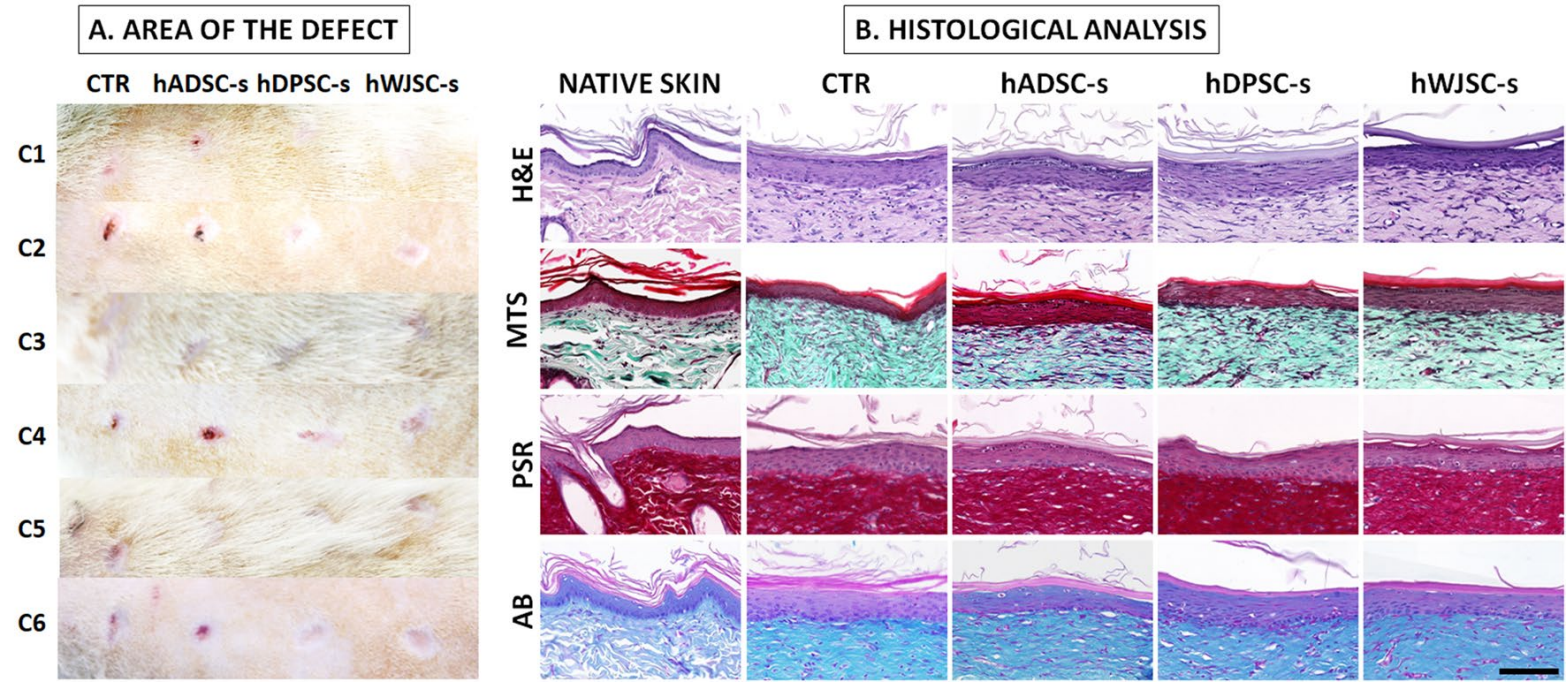
Preliminary in vivo analysis of skin defects treated with each experimental condition after 4 weeks of follow-up. (**A**) Macroscopical images corresponding to each case (C1 to C6). (**B**) Histological analysis using hematoxylin–eosin (H&E), Masson trichrome staining (MTS), picrosirius red (PSR) and alcian blue (AB). Native skin corresponds to histological images of native non-injured skin. *CTR* control group, *hADSC-s* human adipose-derived MSC secretome, *hDPSC-s* human dental pulp MSC secretome, *hWJSC-s* Wharton’s jelly MSC secretome. The scale bar shown on the last histological image applies to all histological images and represents 100 µm.

As shown in Fig. 6A, our preliminary analysis of skin wounds treated with the different secretomes revealed some differences among samples. In the first place, quantification of the area of the skin injuries at the end of the follow-up period showed that the average area of control injuries was 2.49 ± 2.03 mm^2^. In the second place, the analysis of the defects treated with the different secretomes showed that the average area was 2.07 ± 1.81 mm^2^ for hADSC-s, 0.26 ± 0.30 mm^2^ for hDPSC-s and 0.25 ± 0.29 mm^2^ for hWJSC-s. Differences with control were non-significant for hADSC-s (p = 0.8181) and statistically significant for hDPSC-s and hWJSC-s (p = 0.0259 in both cases). No side effects (necrosis, hemorrhage, infection, tumorigenesis, etc.) were observed after secretome treatment.

In addition, our preliminary histological analysis of the skin treated with the different experimental conditions showed very few differences among groups (Fig. 6B). In general, the epidermis found at the regeneration site consisted of a stratified epithelium showing different layers with signs of terminal keratinization and squamation at the most superficial layer, with no differences among the study groups. No differences were found for the presence of collagen fibers and proteoglycans at the dermal layer. As compared to the non-injured native skin of the animal, regenerated skin was devoid of the typical skin annexes found in native skin and the dermo-epidermal junction was rather flat, without the epithelial rete-ridges and dermal papillae of the normal skin. None of the samples showed any pathological findings such as inflammation, malignant transformation or other complications.

## Discussion

The generation of artificial skin substitutes by tissue engineering is challenging^8–10^. However, the prevalence of pathologies affecting the skin, such as cancer, traumatic injuries or infections^4,5,30,31^, makes it necessary to search for improved models of bioartificial skin able to contribute to the treatment of these conditions. In recent decades, several improvements have been described in biofabrication procedures for artificial skin^20,32^. However, the time required to obtain plentiful HSEC cultures remains one of the main limitations in skin tissue engineering.

In the present study we evaluated the potential of different types of secretome to shorten the time needed to establish epithelial cell cultures. As described previously, secretomes contain abundant bioactive factors with marked growth and immunomodulatory properties—factors that can induce anti-apoptotic and pro-survival pathways in several cell types^33,34^. In the field of tissue engineering, the MSC secretome was recently used to induce bone, cartilage, dentin, mucosa and pulp tissue proliferation, maturation and differentiation^35–37^. However, its effect on epithelial cells has been mostly unexplored^16^, and the actions of different types of secretome in skin cells have not been compared.

We first carried out a preliminary protein analysis of each type of secretome. The results suggested that all three types of secretome analyzed here contained several types of protein with roles in cell proliferation and metabolism. We found that hDPSC-s had the highest number of proteins expressed in the secretome, followed by hWJSC-s and hADSC-s. Although the presence of these proteins per se is not demonstrative of the potential of each type of secretome, and the composition of secretomes may vary with time^38^, the fact that certain types of secretome contain higher amounts of several types of protein suggests that their potential role may differ, and we hypothesize that the inductive potential of hDPSC-s may be superior to that of the other two types of secretome. Interestingly, all three secretomes contained detectable amounts of five proteins related to cell proliferation. Among these common proteins, it has been demonstrated that serpin E1 is able to stimulate keratinocyte migration during cutaneous injury repair^39^, while TIMP-1 and thrombospondin-1 have been linked to cell differentiation and migration, acting as growth factors^40,41^. In addition, PTX3 is reportedly involved in cell proliferation in prostate cells and other cell types^42,43^, and serpin F1 was shown to participate in the proliferation of human skin cells^44^. The presence of these relevant proteins in all three secretome studied here supports their use in skin tissue engineering protocols.

Other proteins that play a crucial role in cell viability, proliferation and migration were also present in specific types of secretome, e.g. MMP-9, DPPIV, angiogenin, IL-8, MCP-1 and uPA, which were expressed in hDPSC-s and hWJSC-s. Remarkably, one of the proteins expressed strongly in hDPSC-s was VEGF, which is classically associated with angiogenic roles and was also recently characterized as a keratinocyte growth factor during wound repair^45^. Previous research demonstrated that angiogenic factors such as VEGF can improve cell proliferation, cell migration, and apoptotic inhibition^46^.

We then evaluated the effects of the different types of secretome on cell viability with a two-armed approach to analysis based on LIVE/DEAD assay and DNA quantification protocols. Taken together, our cell viability results help to establish the biosafety levels of secretome treatment used ex vivo, given that cell cultures exposed to different secretomes were free of any detectable deleterious effects in terms of cell viability, as previously reported for different types of secretome and different applications^38,47^. Furthermore, we found that different types of secretome showed a trend toward a positive effect on cell viability. On one hand, our LIVE/DEAD assay results raise the possibility of improving cell survival by using secretome-based culture media. Moreover, this trend was confirmed statistically in DNA quantification assays, and we found that most conditions involving hWJSC-s and hDPSC-s (especially the former) were associated with a significant improvement in cell viability compared to control cells cultured with basal media. These results are in agreement with previous reports suggesting that secretome-based culture technologies can help increase cell survival and reduce cell death ex vivo and in vivo^48,49^. The differences we found between results obtained with the two analytical methods may be explained by their different sensitivities. Future studies with larger sample sizes should be carried out to verify our results. Nonetheless, our preliminary results generally confirm that the use of secretomes, especially hWJSC-s and hDPSC-s, can help increase the survival of HSEC in culture, in light of previously evidence that the use of MSC-derived secretomes is able to increase viability in different types of epithelial cell subjected to experimental damage^50^.

Once cell viability was confirmed, we analyzed the ability of each type of secretome to induce cell proliferation. Our cell quantification results showed that all three types of secretome were able to favor cell proliferation, especially at longer follow-up times (72 and 120 h). Although all secretomes succeeded in yielding high numbers of cells, we found that hDPSC-s tended to be more efficient than hADSC-s or hWJSC-s at 72 h, whereas hWJSC-s was the most efficient at 120 h. The fact that induction was not clearly efficient during the first days of exposure is in agreement with previous reports suggesting that the inductive effect of secretomes may require several days of exposure to become fully effective^38^. This finding may be explained by the occurrence of a cell adaptation period after the conventional medium is replaced with a secretome-containing medium^51,52^. To verify these results, we quantified cell proliferation by determining cell metabolic activity with WST-1 assays. Our results showed that WST-1 activity was increased in some groups, with hDPSC-s tending to show higher activity than hADSC-s and hWJSC-s. The increased inductive potential of hDPSC-s was previously proposed by researchers who suggested potential therapeutic applications of hDPSC-s for hepatic regeneration^53^, and for induced osteogenic and neurogenic regeneration^54^.

Along with cell proliferation, we analyzed the ability of HSEC to expand ex vivo in wound healing tests. Our results confirmed that control HSEC required up to 36 h to proliferate, expand, and completely cover the culture surface; these findings support the need to find alternative culture methods able to enhance the proliferation capacity of human keratinocytes. Nonetheless, we found that all three types of secretome compared in the present study showed potential to reduce the time required for full coverage of the culture surface with HSEC, thus confirming the ability of these products to favor wound healing. In line with the results obtained with proliferation analysis methods, hWJSC-s and hDPSC-s showed the most promising results, especially at the highest concentrations of 75% and 100% after 24 h of follow-up. These outcomes are in agreement with previous studies demonstrating that secretomes derived from bone marrow MSC and adipose tissue MSC can efficiently improve migration in wound healing assays, with 95% of the culture surface covered by proliferating keratinocytes after 27 h of exposure to the secretome^55^. In consonance with these findings, a human MSC secretome was previously shown to improve wound healing and induce cell migration even when keratinocytes were subjected to hypoxia and serum starvation^56^.

To confirm these findings in vivo, we evaluated our secretomes in a small group of animals. Although our results were very preliminary and conclusions cannot be taken at this stage, we first found that no side effects were associated to the treatment, suggesting that secretome treatment could be safe in vivo. In addition, a positive preliminary effect was found regarding epithelization and healing of the skin wounds inflicted in the animals, especially when hDPSC-s and hWJSC-s were used. Interestingly, our preliminary histological analyses support the idea that the use of the different secretomes evaluated in this work could be safe for the treated skin, since no histological differences were found between controls and defects treated with secretome. Both the morphological structure and the presence of fibrillar and non-fibrillar components of the tissue extracellular matrix were similar in all groups. In this regard, it is important to note that previous works demonstrated that MSC may have anti-inflammatory and anti-fibrotic properties and are able to modulate the synthesis of extracellular matrix components^57^. Despite these results should be confirmed in a larger cohort of animals, our results suggest that secretomes could enhance in vivo skin regeneration and wound healing, as previously suggested^58^.

Few studies published to date have been designed to compare the effect of different types of secretome on cell proliferation and migration^14^, and most of these reports focused on evaluating the effects of secretomes derived from bone marrow MSC. In the present study we evaluated three types of secretome derived from different sources of MSC that are relatively accessible and easy to obtain from liposuction procedures, third-molar extractions, or umbilical cords discarded after delivery. These potential sources contrast with bone marrow aspirates, which require a more invasive harvesting procedure that may be associated with considerable morbidity^26,59^. In addition, no previous studies have investigated the effects of increasing concentrations and different types of MSC secretome on cell viability, proliferation, and migration, or on protein expression.

In summary, the present findings suggest that MSC-derived secretomes are not associated to significant side effect and could be used for in vitro and in vivo preclinical approaches. The use of secretome was associated with increased viability, proliferation potential in human skin epithelial cells, suggesting that secretome could be considered as a potential source for future applications in tissue engineering protocols. The fact that induction efficiency was greater with hDPSC-s and hWJSC-s than with hADSC-s may be related to the undifferentiated origin of both types of cell, which originate very early during embryonic development^60–63^. Compared to adipose tissue, bone marrow, or other adult sources of MSC, dental pulp and umbilical cord are thought to contain important cell precursors that remain in an undifferentiated stage^64^, and that have a strong potential for multilineage differentiation^65^. In this connection, hDPSC-s was shown to be potentially useful in regenerative medicine, and this type of secretome is considered to be clinically useful in future therapeutic applications^66^. Our results support the use of these products to improve currently available cell culture protocols applied in tissue engineering of the human skin and other organs that require epithelial cell culture, e.g. the human cornea, oral mucosa, and palate. Although our results support the preferential use of a specific type of MSC to generate secretomes, an important unanswered question is interindividual variation and heterogeneity. In order to control donor-specific differences, we combined the secretomes obtained from three different cell donors in each case, but future studies should determine the influence of donor-specific characteristics on the results of each type of secretome.

## Conclusions

In summary, our findings suggest that MSC-derived secretomes might be used ex vivo without affecting cell viability, and that in vivo use was not associated to significant detectable side effects. Furthermore, the use of this technology could contribute to improve wound healing. However, additional studies will be needed to evaluate the real clinical potential of MSC-derived secretomes.

## Supplementary Information

Supplementary Table 1.

## Data Availability

The datasets generated and/or analyzed during the current study are available from the corresponding author on reasonable request.

## Abbreviations

DMEM: Dulbecco’s Modified Eagle’s Medium
EDTA: Trypsin-ethylenediaminetetraacetic acid
EGF: Epidermal growth factor
FBS: Fetal bovine serum
hADSC: Human adipose derived stem cells
hDPSC: Human dental pulp stem cells
HSEC: Human skin epithelial cells
hWJSC: Human Wharton jelly’s stem cells
MSC: Mesenchymal stem cells
PBS: Phosphate-buffered saline
