# Supplementary material for: Development of secretome-based strategies to improve cell culture protocols in tissue engineering
Source: Sci Rep. 2022 Jun 15;12:10003. doi: 10.1038/s41598-022-14115-y (PMC9200715; doi:10.1038/s41598-022-14115-y)
Supplement: Supplementary file 1 — Supplementary Table 1. [file 41598_2022_14115_MOESM1_ESM.docx]

**Supplementary** **Table 1.** Statistical comparison of all groups with the Kruskal–Wallis test for the results of the LIVE/DEAD® assay, DNA release, cell count (flow cytometry), and WST-1 methods. Statistically significant differences (*p*<0.05) are highlighted in bold.

| **Kruskal-Wallis** | | | | | |
| --- | --- | --- | --- | --- | --- |
|  | | ***24 h*** | ***48 h*** | ***72 h*** | ***120 h*** |
| **LIVE/DEAD® assay** | **25%** | **0.006** | **0.002** | **0.003** | **0.003** |
|  | **50%** | **0.006** | **0.002** | **0.004** | **0.003** |
|  | **75%** | **0.002** | **0.003** | **0.005** | **0.001** |
|  | **100%** | **0.004** | **0.002** | **0.003** | **0.002** |
| **DNA release** | **25%** | **<0.001** | **<0.001** | **0.002** | **0.002** |
|  | **50%** | **0.002** | **0.002** | **0.001** | **<0.001** |
|  | **75%** | **0.001** | **0.003** | **<0.001** | **<0.001** |
|  | **100%** | **0.001** | **0.001** | **<0.001** | **0.002** |
| **Cell count (flow cytometry)** | **25%** | 0.059 | **0.034** | **0.005** | **0.002** |
|  | **50%** | 0.129 | **0.003** | **0.016** | **0.002** |
|  | **75%** | **0.002** | 0.163 | **0.047** | **0.004** |
|  | **100%** | 0.078 | **0.007** | **0.002** | **0.003** |
| **WST-1** | **25%** | **0.034** | **0.022** | **0.003** | **0.012** |
|  | **50%** | **0.008** | **0.026** | **0.272** | **0.002** |
|  | **75%** | **0.007** | **0.004** | **0.008** | **0.021** |
|  | **100%** | 0.105 | **0.042** | **0.004** | **0.002** |
